# Mitoxantrone Induces Natural Killer Cell Maturation in Patients with Secondary Progressive Multiple Sclerosis

**DOI:** 10.1371/journal.pone.0039625

**Published:** 2012-06-29

**Authors:** Coralie Chanvillard, Jason M. Millward, Marta Lozano, Isabell Hamann, Friedemann Paul, Frauke Zipp, Jan Dörr, Carmen Infante-Duarte

**Affiliations:** 1 Experimental and Clinical Research Center, Charité Medical Faculty and the Max-Delbrück Center for Molecular Medicine, Berlin, Germany; 2 Clinical and Experimental Multiple Sclerosis Research Center, Charité-Universitätsmedizin Berlin, Germany; 3 NeuroCure Clinical Research Center, Charité-Universitätmedizin, Berlin, Germany; 4 Department of Neurology, University Medicine Mainz, Germany; Klinikum rechts der Isar der Technischen Universitaet Muenchen, Germany

## Abstract

Mitoxantrone is one of the few drugs approved for the treatment of progressive multiple sclerosis (MS). However, the prolonged use of this potent immunosuppressive agent is limited by the appearance of severe side effects. Apart from its general cytotoxic effect, the mode of action of mitoxantrone on the immune system is poorly understood. Thus, to develop safe therapeutic approaches for patients with progressive MS, it is essential to elucidate how mitoxantrone exerts it benefits. Accordingly, we initiated a prospective single-arm open-label study with 19 secondary progressive MS patients. We investigated long-term effects of mitoxantrone on patient peripheral immune subsets using flow cytometry. While we corroborate that mitoxantrone persistently suppresses B cells *in vivo*, we show for the first time that treatment led to an enrichment of neutrophils and immunomodulatory CD8^low^ T cells. Moreover, sustained mitoxantrone applications promoted not only persistent NK cell enrichment but also NK cell maturation. Importantly, this mitoxantrone-induced NK cell maturation was seen only in patients that showed a clinical response to treatment. Our data emphasize the complex immunomodulatory role of mitoxantrone, which may account for its benefit in MS. In particular, these results highlight the contribution of NK cells to mitoxantrone efficacy in progressive MS.

## Introduction

Multiple sclerosis (MS) is the most common autoimmune disease of the central nervous system (CNS) leading to severe disability in young adults. It is considered to be initiated by autoreactive T cells that recognize CNS antigens, and in concert with numerous immune cells orchestrate an inflammatory reaction which eventually results in demyelination and neuroaxonal damage [1]. The most typical disease course is relapsing-remitting MS (RRMS) characterized by total or partial recovery after attacks. Most patients initially displaying a relapsing-remitting course eventually convert to a secondary progressive disease course (SPMS) after 10–25 years of disease [2].

Current therapies for MS focus mainly on immune aspects of the disease and benefit principally patients with RRMS, while their efficacy is minimal or even lacking in patients with primary progressive disease. Mitoxantrone (MX) is one of the few treatments licensed for use in SPMS [3]. MX is an anti-neoplastic anthracenedione derivative that inhibits DNA replication and induces single and double strand breaks by intercalating in DNA through hydrogen bonding [4]. The mechanisms of action of MX are still not fully understood, and clear data on its effects on the immune system are limited [5]. Although MX has shown effectiveness in SPMS [6], a substantial proportion of patients fail to respond to treatment, and thus there is an urgent need to identify markers that allow the prediction of individual treatment responses. Moreover, the administration of MX is limited to a treatment-period of 2–3 years by the cumulative dose-dependent risk of severe adverse effects, such as cardiotoxicity [7]–[11] and potential leukemia development [12]. Nevertheless, understanding how MX benefits SPMS patients is essential for establishing safer and more effective treatments for this group of patients. Therefore, we conducted a longitudinal study on an SPMS cohort, analyzing intra-individual comparisons (baseline versus treatment) of major populations of peripheral blood lymphocytes using flow cytometry.

## Methods

### Study Design and Participants

A prospective monocentric single-arm open-label study design was used to evaluate the effects of MX treatment on immunological parameters in MS patients. The study was approved by the ethics committee of the Charité – University Medicine Berlin and was conducted in accordance with the Declaration of Helsinki, the guidelines of the International Conference on Harmonization of Good Clinical Practice, and the applicable German laws. All participants gave informed written consent. Patients were screened and enrolled at the neuroimmunology outpatient clinic of the Charité – Universitaetsmedizin Berlin in Germany. Inclusion and exclusion criteria are summarized in Table 1. Patients received MX according to the standard protocol [6], meaning that unless dose reduction was required owing to side effects such as hematological abnormalities, an MX dose of 12 mg/m^2^ body surface area (BSA) was applied intravenously every three months up to a cumulative dose of 140 mg/m^2^ BSA. Treatment with other cytotoxic or immunomodulatory drugs was prohibited during the study.

**Table 1:** Inclusion and exclusion criteria of the study.

**Inclusion criteria**	• definite diagnosis of MS according to the 2001 McDonald criteria
	• active disease defined by at least two relapses and/or progression by at least one point on the expandeddisability status scale (EDSS) in the preceding 18 months which was not sufficiently controlled by interferon betaor glatiramer acetate
	• EDSS score of 3–6.5
	• at least two months free of treatment with interferon-beta or glatiramer acetate
	• no relapse or steroid treatment four weeks prior to enrolment
	• no prior treatment with immunosuppressants
**Exclusion criteria**	• pregnancy or breast feeding
	• cardiac disease
	• severe leucopenia
	• inability to provide informed consent

In this study, we aimed to determine the persistent effects of MX that may account for its long-term benefit in SPMS. Therefore, study-related clinical assessment and blood sampling for evaluation of immunological parameters was done immediately before the next subsequent MX administration, i.e. three months after the previous MX-infusion. Clinical examination and venipunction occurred at baseline, after six months (directly prior to the third MX cycle) and after twelve months (directly prior to the 5th MX cycle). As controls, we included 10 RRMS patients with no sign of disease activity (mean EDSS of 2), as well as 8 healthy controls. Both groups were gender and age matched.

### Reagents

Human Fc fragment was purchased from Calbiochem (Merck Chemicals Ltd., Darmstadt, Germany). The following antibodies were used: against CD3 (AlexaFluor700), CD16 (Pacific Blue), CD62L (APC), CD4 (PerCP), CD8 (FITC), CD14 (FITC), CD19 (PE) from Becton-Dickinson (Heidelberg, Germany); CD56 (FITC), CX3CR1 (PE) from MBL (Nagoya, Japan); CD27 (PE-Cy7) from eBioscience (San Diego, CA); CD57 (APC), NKp30 (APC), NKp46 (APC), KIR2DL1/2DS1, KIR3DL1/3DL2 (APC) from Miltenyi Biotech (Bergisch-Gladbach, Germany); CD94/NKG2A (PerCP) and KIRDL2/2DS2/2DL3 (APC) from R&D KIR3DL1/3DS1 (APC) from Beckman Coulter (Krefeld, Germany).

### Isolation of PBMCs

Peripheral blood mononuclear cells (PBMCs) were obtained from heparinized peripheral blood from the patients and isolated by density gradient centrifugation (Percoll, Nycomed Pharma, Roskilde, Denmark) according to the manufacturer’s instructions. PBMC were then cryopreserved in liquid nitrogen for later analysis.

### Flow Cytometry Analysis

For *ex vivo* investigation, whole blood was lysed, washed, and stained with antibodies against CD14, CD19 or CD3/CD4/CD8 to identify monocytes, B cells or T cells, respectively. Neutrophils were identified based on gating on the granulocyte population in the forward and sideward scatter profile, and CD16 positivity.

For the phenotype characterization of natural killer (NK) cell subpopulations, thawed PBMCs were washed and incubated with antibodies against CD3, CD56, CD16, CX3CR1, CD62L, CD57, CD27, CD94/NKG2A, NKp30, NKp46, and panKIR (KIR2DL1/2DS1/2DL2/2DS2/2DL3/3DL1/3DL2/3DS1) as described previously [13]. Absolute cell numbers were determined with the use of TrueCount beads (BD). Data were analyzed using the FACS DIVA software.

### Statistical Analysis

The paired t-test was used to calculate p-values for comparisons between two groups (i.e. baseline versus six months and baseline versus 12 months). Repeated measures ANOVA was used for comparisons between three groups (i.e. baseline versus six and 12 months), with the Tukey post-hoc test. Statistical significance was defined as p<0.05, and depicted as *p<0.05; **p<0.01; ***p<0.001. One-way ANOVA with the Bonferroni post-hoc test was used to compare baseline and 12 months of treatment with healthy controls and with RRMS patients. We verified that the data conformed to a Gaussian distribution. Statistical significance was depicted as # p<0.05; ### p<0.001.

## Results

### Cohort Description

Of the 19 SPMS patients screened, 15 patients were included in this study because four patients did not conform to the eligibility criteria. From the 15 patients enrolled, two patients dropped out before receiving the third MX dose because of intolerability, and one patient terminated MX treatment before receiving the 5^th^ dose because of severe disease progression. The remaining twelve patients completed the study period of twelve months.

In accordance with the pivotal MX trial in MS [6], clinical assessment of therapy response was based primarily on the EDSS and secondarily on the occurrence of relapses. Those patients who improved or remained stable on the EDSS throughout the study period and who did not experience any relapses were considered treatment responders; patients who deteriorated on the EDSS, experienced a new relapse or both where classified as non-responders. Altogether, five patients were classified as treatment responders and eight patients as non-responders to MX treatment. The demographic and clinical features of these patients are summarized in table 2.

**Table 2:** Clinical data of the 15 SPMS patients included in the study.

Patients	Sex	Age (years)		Disease duration (months)	EDSS				Relapses	Response
		Start MX	Disease onset		Base line	Month 6	Month 12	Month 18		
1	m	42	36	69	4.0	4.0	4.0	4.0	no	R
2	m	44	39	57	3.5	3.0	3.0	3.0	no	R
3	m	34	31	36	5.5	6.0	6.5	6.5	yes (2x)	NR
4	f	55	36	225	6.5	6.5	nd	nd	no	na
5	m	41	37	46	5.5	6.0	6.0	6.5	no	NR
6	m	49	32	207	5.5	5.5	5.5	nd	yes	NR
7	m	30	18	144	5.5	5.5	5.5	5.5	no	R
8	f	35	30	65	3.5	3.5	4.0	4.0	no	NR
9	m	40	23	204	5.5	6.0	6.0	nd	no	NR
10	f	51	46	55	4.0	4.0	4.5	4.5	no	NR
11	m	53	43	120	4.0	5.5	nd	nd	no	NR
12	m	66	46	246	6.5	7.0	7.0	nd	no	NR
13	m	37	26	133	6.5	6.5	7.0	6.5	no	R
14	f	57	35	263	5.0	5.0	4.0	4.0	no	R
15	m	52	32	240	6.0	6.0	nd	nd	no	na

nd, not determined; R, responder; NR, non-responder; na, not available.

### Effects on MX Treatment on the Frequency of Peripheral Immune Cell Populations

**Figure 1:**
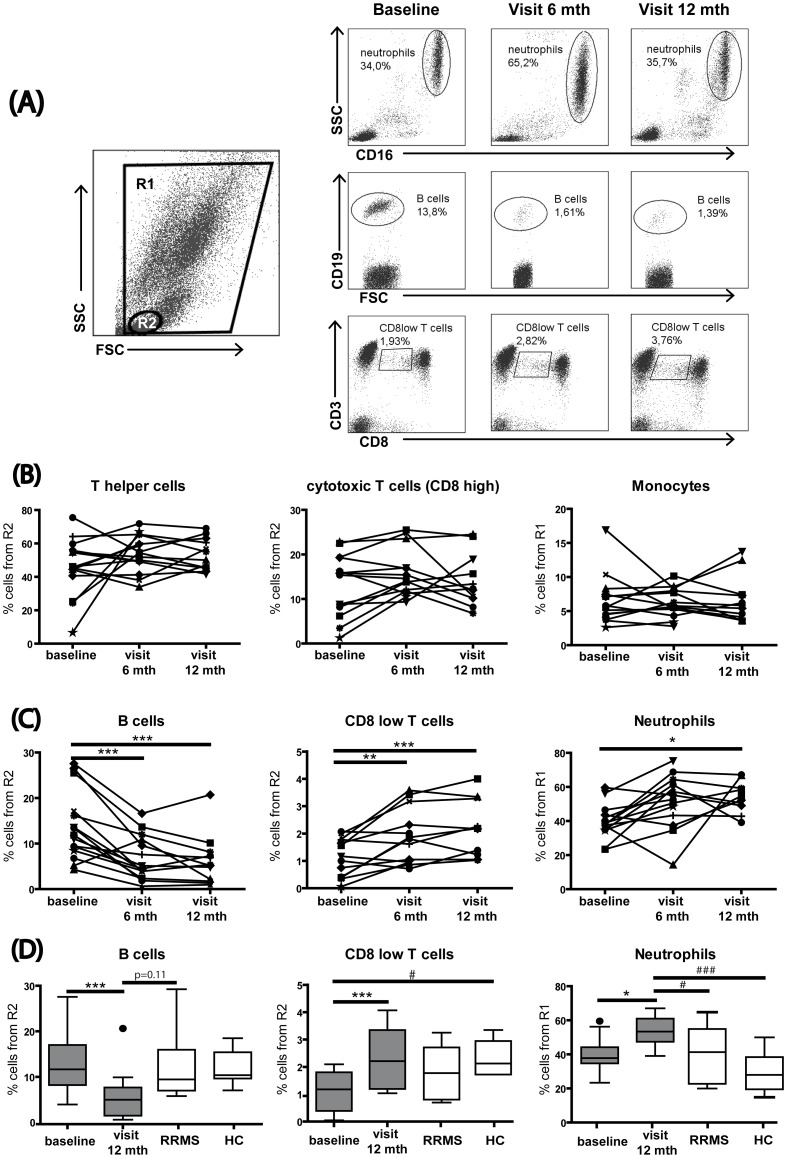
MX treatment leads to a persistent reduction of B cells and enrichment of neutrophils and CD8^low^ T cells. Peripheral blood samples from SPMS patients were surface stained for CD16, CD19, CD14, CD3, CD4, and CD8. (A) A representative flow cytometry dot plot shows the gating strategy for leucocytic populations: R1, comprising all cells without the debris and R2, representing the lymphocytes. Representative flow cytometry plots showing MX-induced changes over time in neutrophils, B cells and CD8^low^ T cells. (B) The T helper cell (CD4+), the cytotoxic T cell (CD8^high^), and the monocyte (CD14+) populations did not change over time. (C) The B cell (CD19+) population decreased significantly after six months and 12 months of treatment. The cytotoxic T cell (CD8^low^) population increased significantly after six months and 12 months of treatment, and the neutrophil (CD16+) population was enriched after 12 months of treatment. (D) Comparison of frequencies of B cells, CD8^low^ cells and neutrophils before and after treatment and in patients with stable MS and healthy individuals. B cell levels presented no difference. CD8^low^ T cell levels after 12 months of treatment were normalized to healthy controls levels. Neutrophil levels after 12 months of treatment were increased compared to the controls. Repeated-measures ANOVA *p<0.05; **p<0.01; ***p<0.001; One-way ANOVA #p<0.05; ###p<0.001. FSC, forward scatter; SSC, side scatter; mth, months.

In order to apprehend the persistent effects of MX on the immune system in SPMS patients, we first determined the effect of the treatment on neutrophils, monocytes and T and B lymphocytes in whole blood, directly after venipunction at baseline and three months after six months and 12 months of treatment (Figure 1).

MX treatment did not affect the populations of CD14^+^ monocytes, CD4^+^ Th or conventional CD8^high^ T cells at these time points (Figure 1B). In contrast, we observed a significant increase of a subset of immunomodulatory CD8^low^ T cells at six and 12 months (repeated measures ANOVA, p  = 0.0002) as well as an increase in the frequency of neutrophils at month 12 (repeated measures ANOVA, p  = 0.044, Figure 1C). Moreover, confirming previously reported data, the B cell population was persistently reduced during the entire observation period (repeated measures ANOVA, p<0.0001, Figure 1C). Furthermore, in order to elucidate if MX-induced alteration of the proportion of B cells, CD8^low^ T cells and neutrophils reflected a restoration toward normal cell levels observed in healthy individuals or to levels observed in stable patients, we assessed the percentages of these three immune cell populations in a gender and age-matched cohort of RRMS patients with mild and stable disease, and in a matched group of healthy controls. Figure 1D shows that only in the case of the immunomodulatory CD8^low^ T cell population, MX treatment seems to restore the proportion of these cells to levels observed in healthy controls (one-way ANOVA p  = 0.015). No significant difference was observed between frequencies of CD8^low^ T cells before or after MX application and the levels observed in stable MS patients. In contrast, the MX-induced neutrophil enrichment does not seem to reflect any trend towards normalization since the proportion of neutrophils at 12 months was also significantly elevated, compared to both healthy controls and stable MS patients (one-way ANOVA p  = 0.001). Likewise, the selective depletion of B cells during MX treatment cannot be considered as a normalization of immune cell proportion, at least in terms of absolute numbers.

We also examined the effects of MX on NK cells in fresh blood and observed an increased frequency of NK cells at six months. In light of this observation, we conducted a more detailed analysis of these cells in subsequent investigations using frozen material, in an attempt to clarify specifically how the NK cell compartment is modulated during MX treatment.

### Effects of MX Treatment on Frequency and Absolute Numbers of Circulating NK Cells

**Figure 2:**
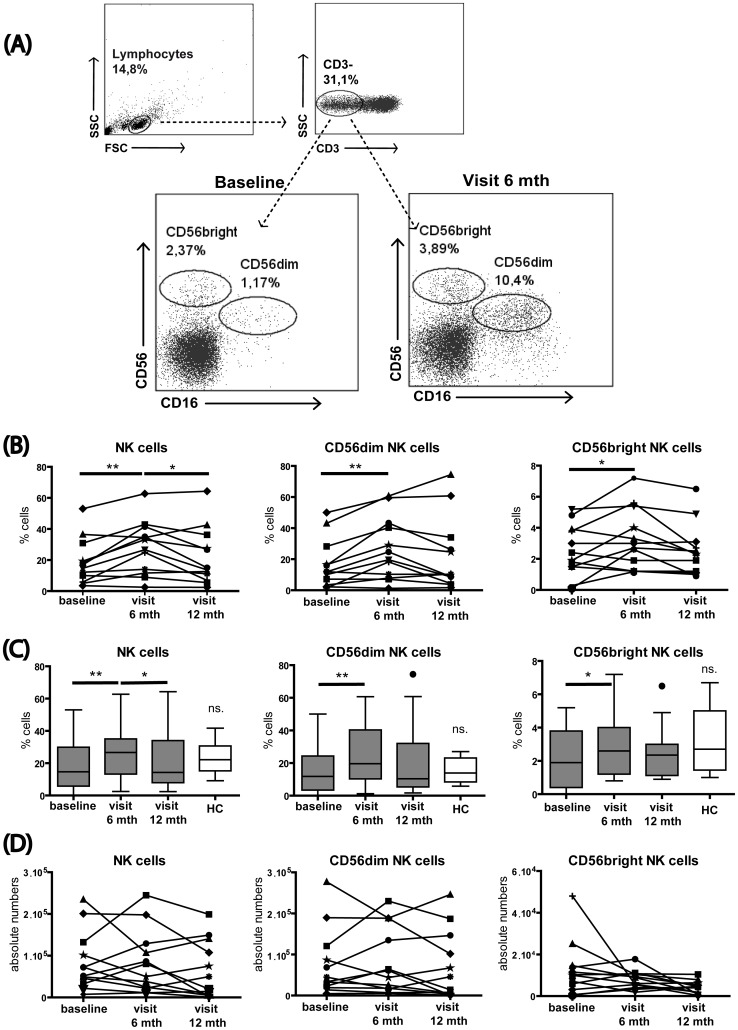
MX treatment promotes NK cell enrichment. Thawed PBMCs from SPMS patients were stained for NK cells and major subsets using CD56 and CD16. (A) Representative flow cytometry plots show the NK cell gating strategy and MX-induced changes over time in NK cells. (B) Shows the population percentages. After six months of treatment, the NK cell population was significantly enriched and then decreased from six to 12 months of treatment. The CD56^dim^ and CD56^bright^ NK cells subsets were significantly increased after six months of treatment, but no difference was detected from six to 12 months. (C) Frequencies of NK cells and NK cell subsets in SPMS patients before and after MX treatment compared to the frequency observed in matched healthy individuals. (D) Shows absolute counts of NK cells and CD56 subsets: NK cells, CD56^dim^ and CD56^bright^ NK cell subsets remained unchanged over time. *p<0.05; **p<0.01; FSC, forward scatter; SSC, side scatter; mth, months; ns. non significant.

We then analyzed NK cells at the different time points using thawed PBMCs. NK cells were first categorized according to their expression of CD56 and CD16 in the well defined subsets of CD56^dim^ and CD56^bright^ NK cells (Figure 2A). Using a more comprehensive set of markers, we could confirm a significant increase of the total NK cell frequency during the first part of the study, i.e. after six months of treatment (Figure 2B). This initial increase receded in the latter stages after 12 months of treatment (repeated measures ANOVA, p  = 0.001). Also here, we examined whether MX effects on NK cells reflected a restoration toward NK cell frequencies observed in healthy individuals. As shown for B cells and neutrophils, MX treatment seems not to restore the proportion of NK cells or their subsets to levels observed in healthy controls (Figure 2C). Interestingly, we did not observe any increase of the absolute NK cell numbers during treatment, indicating that the increased frequency was rather an indirect cell enrichment due to the depletion of other major immune cell populations (Figure 2D).

### Effects of MX Treatment on Maturation and Differentiation of Circulating NK Cells

Next, we focused on the effects of MX on the distinct NK cell subsets, the cytotoxic CD56^dim^ and immunomodulatory CD56^bright^ NK cells. The frequency of both CD56^dim^ and CD56^bright^ NK cells increased concomitantly after six months of treatment (Figure 2B) (repeated measures ANOVA, p = 0.004 and 0.029, respectively). Again the analysis of absolute cell numbers did not show any statistically significant differences in these populations (Figure 2C) confirming the interpretation of a general and non-specific enrichment of all NK cells at six months. Moreover, neither total NK cells nor CD56^dim^ and CD56^bright^ NK cell evolution after treatment presented normalization to the healthy control levels (Figure 2C). We and others have recently reported on different stages of NK cell subsets, cell maturation with distinct functional properties that can be characterized by the expression of specific markers including CD27 [14], CD57 [15], CD62L [16], or CX3CR1 [13].

**Figure 3:**
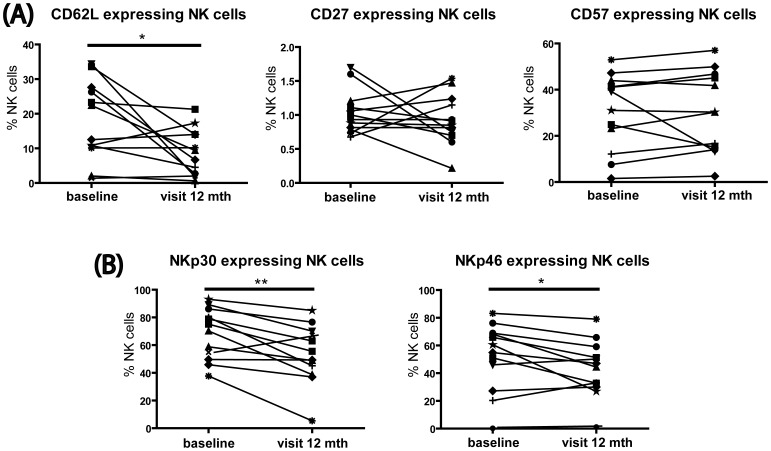
MX treatment induces NK cell maturation. Thawed PBMCs from MX-treated SPMS patients were stained for expression of CD62L, CD27, CD57, NKp30, and NKp46 on NK cells. (A) Following 12 months of treatment, the population of cells expressing the maturation marker CD62L decreased, but no change was detected for CD27 and CD57 expressing NK cells. (B) The population of cells expressing the activatory receptors NKp46 and NKp30 showed a significant decrease after 12 months of treatment. *p<0.05; **p<0.01; mth, months.

To determine the effects of MX on the NK cell maturation and activation phenotype, we analyzed an array of NK cell markers at months six and 12 of treatment. Although at month six the NK cell phenotype appeared not to be affected, we did observe a long-term MX-associated reduction of CD62L expression (p  = 0.025), which is indicative of a process of maturation [16]. The expression of CD27 and CD57 remained unaltered (Figure 3A).

Moreover, to prove that this maturation process is coupled to the regulation of NK cell receptors, we examined the expression of the activatory receptors NKp30 and NKp46, and the inhibitory receptors CD94/NKG2A and panKIR, which are known to be regulated during NK cell maturation [13]. Our data show that, coinciding with maturation, both NKp46 (p = 0.041) and NKp30 (p = 0.004) were decreased after treatment (Figure 3B). No changes were detected in the inhibitory receptors CD94/NKG2A and killer cell immunoglobulin-like receptors (panKIR) (data not shown). Thus, altogether these results indicate that MX promoted a shift towards a more mature NK cell phenotype, as reflected by the downregulation of CD62L as well as NKp46 and NKp30. However, these changes in NKp30, NKp46 and CD62L expression did not reflect any trend toward a restoration to healthy control levels (data not shown).

### Association of the Clinical Response to MX and the Modulation of NK Cells

**Figure 4:**
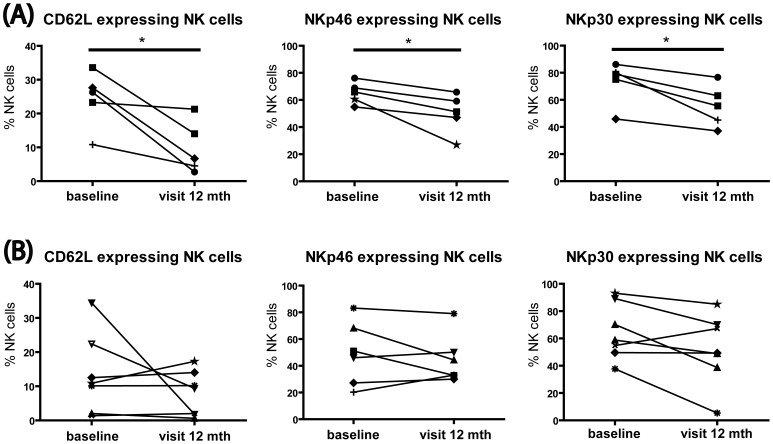
MX-induced NK cell maturation is associated with the clinical response. Thawed PBMCs from SPMS patients stratified into responders and non-responders to MX, were stained for expression of CD62L, CD27, CD57, NKp30, and NKp46 on NK cells. (A) Following 12 months of treatment the responder cohort showed decreased CD62L, NKp46 and NKp30 expression on NK cells. (B) The non-responder cohort did not show any difference in CD62L, NKp46 and NKp30 expression. *p<0.05; mth, months.

In Figure 3, we show a heterogeneous picture on NK cell modulation by MX. Since regulation of NK cells has been associated with response to diverse MS therapies such as daclizumab or interferon beta therapy [17], [18], we asked whether changes in NK cell status may correlate with the treatment response. The cohort of patients was stratified into responders and non-responders to treatment, according to the response criteria described above. Even despite the limited sample size, we demonstrate that maturation, reflected by the downregulation of CD62L (p = 0.029), NKp46 (p = 0.032) and NKp30 (p = 0.020), occurred exclusively in the cohort of responders (Figure 4A). In contrast, patients that did not benefit clinically from MX treatment showed no significant alterations of the various NK cell markers examined here (Figure 4B).

## Discussion

To address the specific and persistent effects of MX treatment on different immune cell subpopulations in SPMS patients, we conducted a longitudinal study on a cohort of 15 SPMS patients. Using flow cytometry, we compared intra-individually (baseline versus treatment) major populations of peripheral blood lymphocytes including NK cells. We demonstrated that, apart from being cytotoxic for B lymphocytes, MX promoted the enrichment of peripheral neutrophils as well as of subsets of CD8^low^ T lymphocytes and of NK cells. In addition, we observed that sustained MX treatment induced a shift in the maturation of circulating NK cells which was associated with clinical response to MX treatment.

Our initial results addressed the effects of MX treatment on major blood cell populations (Figure 1). We showed a dramatic depletion of B cells following MX treatment. The baseline B cell levels in these patients were comparable to levels seen in stable MS patients and healthy controls (Figure 1D), therefore the MX-induced depletion cannot be considered as a normalization of an atypical elevated proportion of B cells, at least in term of quantity. B cell depletion by MX was previously reported by other groups both as an immediate and a persistent consequence of the MX therapy *in vivo* [19]. Moreover, Chan et al. demonstrated that already 1 h after MX infusion not only B cells but also CD8^+^ T cells underwent apoptosis [20]. We did not observe any effects of MX on T cells, which is consistent with the results reported by Gbadamosi et al. [19]. Thus, the immediate MX-induced apoptosis of CD8^+^ T cells is probably a transient phenomenon. However, we observed a strong and consistent increase in the frequency of a subset of CD8 T cells characterized by a lower expression of the CD8 co-receptor.

CD8^low^ T cells are less cytotoxic than CD8^high^ T cells, and they express IL-4, IL-10, and interferon-gamma [21]. CD8^low^ lymphocytes, and in particular CD8^low^ NK cells have been shown to be reduced in untreated patients with clinically isolated syndrome and MS [22]. Interestingly, MX appears to restore the frequency of CD8^low^ T cells to the levels observed in healthy individuals (Fig. 1D), suggesting that this regulation may contribute to the efficacy of the treatment in MS. In any case, it is evident that investigations on the role of CD8^low^ T lymphocytes in MS and other neuroimmunological disorders and their modulation during treatment are worthwhile topics for future investigations.

We also observed an increased frequency of peripheral neutrophils in MX-treated patients, the proportion of which is elevated even when compared to control stable patients or to healthy individuals (Fig. 1D). This was an unexpected result, as MX was reported to normalize IL-6 production [23], a cytokine that together with G-CSF is known to induce neutrophil production [24]. Neutrophil infiltration into the CNS has been associated with the acute EAE phase [25] and with early axonal pathology in EAE [26]. A recent report indicates that neutrophils may also contribute to MS pathogenesis, as patients display elevated numbers of pre-activated or primed neutrophils in peripheral blood 27. It is not clear whether neutrophil activity or phenotype are altered by MX treatment. Since an elevated frequency of neutrophils was observed in both responders and non-responders (data not shown), one may speculate that the observed effect was not related to the efficacy of MX treatment. Similarly, the elevated frequency of CD8^low^ T cells did not appear to be associated with clinical response to MX.

In our study, MX induced an enrichment of the NK cell population after six months of treatment, which subsequently stabilized by month 12 (Figure 2). This was an unexpected finding in view of the potent immunosuppressive function of MX on other lymphocyte populations [28]–[30].

To better understand how MX treatment influences NK cell phenotype, we investigated the treatment effects on NK phenotype and activity. We showed that at six months of treatment, both the cytotoxic CD56^dim^ and the immunomodulatory CD56^bright^ subpopulations were enriched, without any shift to a particular phenotype. Moreover, MX seems not to restore the frequency of NK cells and their subsets to healthy control levels as shown in Figure 2C. Elevated frequencies of total NK cells or of the CD56^dim^ and CD56^bright^ subsets were not accompanied by an elevation of absolute NK cell numbers. Thus, NK cell enrichment seemed to be the indirect consequence of the dramatic suppression of other immune populations, primarily B cells (Figure 1).

Interestingly, prolonged MX treatment did not further affect immune cell frequencies, but appeared to modulate immune cells in a more specific way. In particular, we observed a significant reduction in the frequency of circulating immature NK cells after 12 months of treatment. During maturation, NK cells downregulate the expression of CD27, CD57 and CD62L [16] as well as the expression NKp30 and NKp46 activatory receptors and the inhibitory CD94/NKG2A receptor complex. Here, we showed that at 12 months of treatment, the maturation marker CD62L and the activatory receptors NKp46 and NKp30 were significantly downregulated, suggesting a late and persistent effect of MX on the maturation of circulating NK cells. This could be in part the consequence of the expected elevated susceptibility of immature proliferating NK cells to MX-induced cytotoxicity. Indeed, we previously demonstrated that immature NK cells proliferate much better than mature NK cells in response to IL-2. [13]. However, that cytotoxicity alone may not entirely explain this rather late effect of the treatment, which manifested only after repeated applications of MX, at 12 months. It was previously reported that MX treatment enhances the expression of Th2-related cytokines [31]. IL-4 is known to induce NK cell maturation [32]. Therefore, we speculate that MX promoted enhancement of IL-4 production may also contribute to NK cell maturation in treated patients. Similarly, in agreement with the report of Kienzle et al. on the generation of CD8^low^ T cells in the presence of IL-4 [33], the strong IL-4 production induced by MX may contribute to the expansion of CD8^low^ T cells observed in our study.

In MS, deficient NK cell activity has been extensively reported by several groups during the last 40 years [34]–[37]. Thus, the observed increased frequency of mature and active NK cells may contribute to the benefit of MX in patients with MS, although we could verify that MX treatment did not restore NK cells to a maturation or activation status observed in healthy donors.

Treatment-related enrichment of particular NK cell subsets, or induction of NK cell activation has been associated with the therapeutic success of numerous MS drugs including interferon-beta, glatiramer acetate, and daclizumab [17], [18], [38]–[40]. Stratifying our data according to MX response revealed that the elevated frequency of mature NK cells was observed exclusively in patients that responded to the therapy (Figure 4). This result is especially promising, considering the robust significance seen in this relatively limited data set. Moreover, of all the effects of MX we observed here, only the NK cell effects were related to the clinical response to MX. It remains elusive why NK cell maturation occurred primarily in patients that responded to the treatment. The fact that both responder and non-responder patients showed the same ratio of immature/mature peripheral NK cells at baseline suggests that baseline differences between the two patient groups likely did not account for this effect. Thus far, we have not explored additional factors, such as selective production of Th2-cytokines [31] or selective alteration of the functionality of antigen-presenting cells [41], which may contribute directly or indirectly to NK cell maturation, and which may also differ in responders and non-responders.

Thus, while alteration of immune cell frequencies in the peripheral blood was an early and sustained effect of MX in MS patients, the alteration of NK cell phenotype appears to be a later mechanism of action (observed only after 12 months of treatment), related to the clinical benefits of the treatment.

In conclusion, we have shown for the first time that the NK cell population is promoted by MX treatment *in vivo,* accompanied by a shift towards a mature NK cell phenotype associated with the response to the therapy. The contribution of NK cells to beneficial effects of MX in SPMS may serve as a first step to establish novel and safe treatments for progressive MS.
